# Partial Least Squares Discriminant Analysis and Bayesian Networks for Metabolomic Prediction of Childhood Asthma

**DOI:** 10.3390/metabo8040068

**Published:** 2018-10-23

**Authors:** Rachel S. Kelly, Michael J. McGeachie, Kathleen A. Lee-Sarwar, Priyadarshini Kachroo, Su H. Chu, Yamini V. Virkud, Mengna Huang, Augusto A. Litonjua, Scott T. Weiss, Jessica Lasky-Su

**Affiliations:** 1 Channing Division of Network Medicine, Brigham and Women’s Hospital, Boston, MA 02115, USA; hprke@channing.harvard.edu (R.S.K.); remmg@channing.harvard.edu (M.J.M.); klee-sarwar@bwh.harvard.edu (K.A.L.-S.) reprk@channing.harvard.edu (P.K.), rechu@channing.harvard.edu (S.H.C.), YVIRKUD@mgh.harvard.edu (Y.V.V.); remhu@channing.harvard.edu (M.H.), augusto_litonjua@urmc.rochester.edu (A.A.L.); restw@channing.harvard.edu (S.T.W.); 2 Harvard Medical School, Boston, MA 02115, USA; 3 Division of Rheumatology, Immunology and Allergy, Brigham and Women’s Hospital, Boston, MA 02115, USA; 4 Department of Pediatrics, Massachusetts General Hospital for Children, Boston, MA 02114, USA; 5 Division of Pediatric Pulmonary Medicine, Department of Pediatrics, University of Rochester Medical Center, Rochester, NY 14642, USA

**Keywords:** Partial Least-Squares Discriminant analysis, Bayesian networks, asthma, arginine metabolism, overfitting

## Abstract

To explore novel methods for the analysis of metabolomics data, we compared the ability of Partial Least Squares Discriminant Analysis (PLS-DA) and Bayesian networks (BN) to build predictive plasma metabolite models of age three asthma status in 411 three year olds (*n* = 59 cases and 352 controls) from the Vitamin D Antenatal Asthma Reduction Trial (VDAART) study. The standard PLS-DA approach had impressive accuracy for the prediction of age three asthma with an Area Under the Curve Convex Hull (AUCCH) of 81%. However, a permutation test indicated the possibility of overfitting. In contrast, a predictive Bayesian network including 42 metabolites had a significantly higher AUCCH of 92.1% (*p* for difference < 0.001), with no evidence that this accuracy was due to overfitting. Both models provided biologically informative insights into asthma; in particular, a role for dysregulated arginine metabolism and several exogenous metabolites that deserve further investigation as potential causative agents. As the BN model outperformed the PLS-DA model in both accuracy and decreased risk of overfitting, it may therefore represent a viable alternative to typical analytical approaches for the investigation of metabolomics data.

## 1. Introduction

Asthma is a complex chronic disease, estimated to affect more than 300 million people worldwide, with the majority of cases originating in early life [1]. While many genetic and environmental influences have been characterized [2], the exact etiology of asthma and its trajectory throughout the life-course is still not perfectly understood [3]. Identifying asthma and associated wheeze phenotypes early in life could help to initiate appropriate treatment as early as possible and mitigate the effects on lung function and growth associated with progression of disease.

Advances in high-throughput technologies provide novel tools for the identification of biomarkers of disease, while simultaneously providing new insights into underlying pathogenesis. Metabolomics, which aims to identify and quantify the small molecules (<10 kDa) in a biological sample and is the “ome” most closely related to phenotype, offers a particularly promising approach. Metabolites are involved in all the biochemical reactions regulating the functions of a cell, and represent the downstream products of both the genome and its interactions with the environment [4]. Metabolomics has previously been utilized to identify novel biomarkers of common chronic complex diseases [5]. Furthermore, existing metabolomic studies of asthma have shown promising results. However, accurate prediction or diagnosis of asthma using metabolomic profiles is not yet a reality [6].

Partial Least Squares Discriminant Analysis (PLS-DA) [7] is one of the most commonly used methods in the metabolomics literature, primarily for the assessment of the discriminatory and predictive ability of metabolites and metabolite profiles for diseases and phenotypes [8]. Despite their common use, there are several limitations to PLS-DA models, most notably the issue of overfitting [9,10]; i.e., the generation of models that too closely fit the existing data. Therefore, in addition to identifying real disease discriminators, the PLS-DA models may also explain random variability in the dataset that has no biological relevance to the disease of interest. Unsurprisingly, such overfit models tend to demonstrate limited generalizability, and are difficult to replicate in independent studies.

Consequently, investigators are increasingly looking to alternative approaches [11]. Here we consider one such alternative approach, Bayesian networks (BN). A Bayesian network is a graphical model of the statistical interactions between random variables in a data set, based on Bayesian statistics [12]. These “random variables” are typically metabolites, demographic and clinical covariates, and some phenotype(s) of interest, although in principle any data can be used. The BN then is a directed acyclic graph where nodes represent random variables, and directed edges between nodes represent a statistical dependence of the sink node on the source node. This dependence is determined by a Bayesian likelihood ratio test, called a Bayes Factor [13]. The process of building a BN relies on heuristic algorithms for identifying desirable models, as well as techniques more common in metabolomic analysis including cross-validation and permutation testing. BNs have several desirable properties for metabolomic analysis. (1) BNs have the potential to better capture the relationships between metabolites and a phenotype of interest, through the explicit modeling of nonlinear interactions; (2) BNs include mechanisms which mitigate overfitting, including Bayesian prior probability distributions used in the Bayes Factor tests which bias toward no association; (3) A BN model can be used to identify important individual metabolites and to predict the disease outcome from a subset of available metabolites [14]. We have previously demonstrated the power of Bayesian networks when applied to metabolomic data [15]. In this current study, we assessed and compared the ability of PLS-DA and a metabolomic Bayesian network to predict asthma status at age three in children from the Vitamin D Antenatal Asthma Reduction Trial (VDAART) [16].

## 2. Results

**Figure 1:**
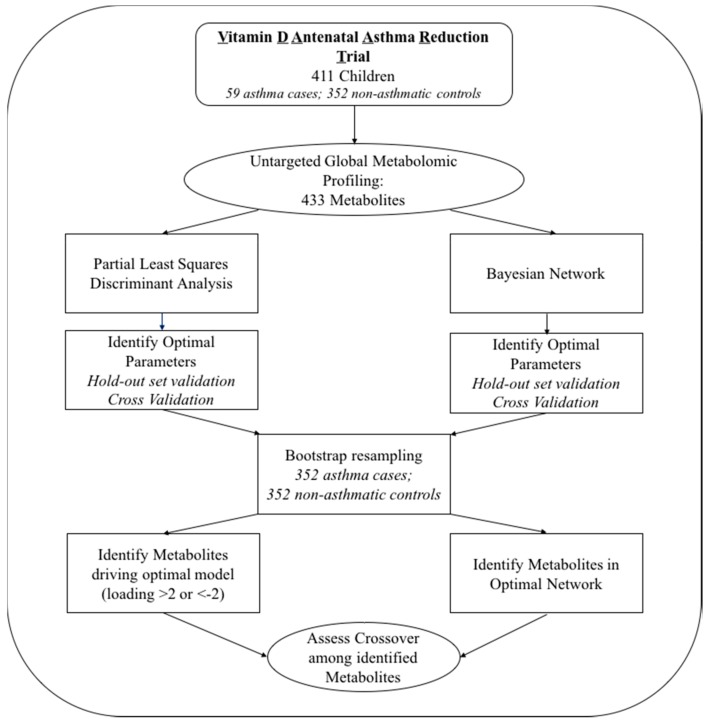
Study Schematic.

The study schematic is outlined in Figure 1. To maximize the value of our limited data, we first used cross-validation to identify optimal parameters for the classifiers. We then used those parameters to build a model using all of the data. This allows us to utilize the greatest amount of available data to obtain the most accurate classifier possible. To check for overfitting, we then used a permutation test.

### 2.1. Study Population

A total of 411 children from the Vitamin D Antenatal Asthma Reduction Trial [16] (VDAART) with mass-spectrometry-based metabolomic profiling on blood samples extracted at age three were included in these analyses. By the age three visit, 59 of the children had been defined by a physician as asthma cases; 352 did not have a physician-diagnosis of asthma during the same period and were considered as controls.

Children with asthma were statistically more likely to be Black (*p* = 0.003), but there were no other significant differences between cases and controls (Table 1). Due to its cohort design, VDAART did not contain an equal number of asthma cases and asthma-free controls. Since strongly unbalanced datasets are a problem for most machine learning classification algorithms [17], leading to algorithms that are biased toward the majority class, we used bootstrap resampling to obtain a balanced dataset [18]. By randomly resampling from the asthma cases with replacement, we obtained a final bootstrap-balanced dataset of 704 subjects with 352 cases and 352 controls (see Section 4). Measurements of 481 known metabolites were available in blood plasma samples collected at age three years for these subjects. Of these, 433 metabolites passed the QC and data processing pipeline and were included in the analyses. The majority of metabolites were lipids (*n* = 142) and amino acids (*n* = 141). Nucleotides, peptides, cofactors and vitamins, energy metabolites, carbohydrates and 57 xenobiotics were also among the measured metabolites (Table S1).

### 2.2. Partial Least Squares Discriminant Analysis

**Figure 2:**
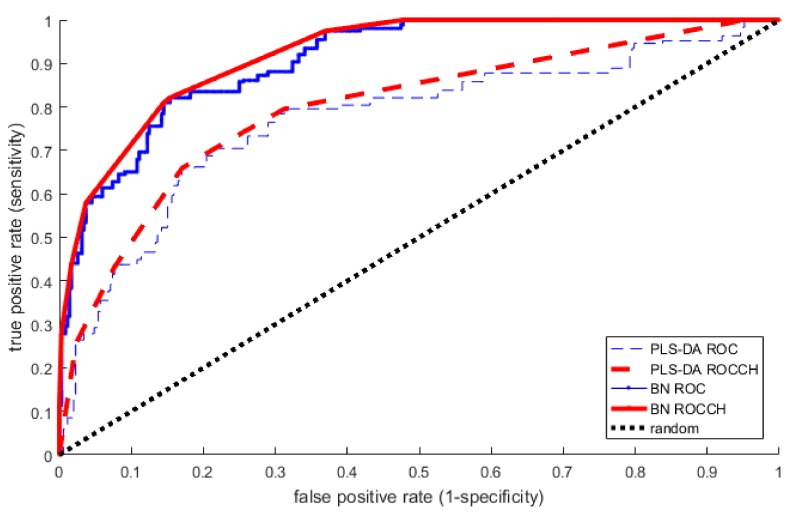
Discrimination of asthma at age three based on plasma metabolomic profiles by PLS-DA (AUCCH = 0.810) and by Bayesian network (AUCCH = 0.921) on the full dataset. AUCCH–Area under the Convex Hull of the Receiver Operator Characteristic curve; BN–Bayesian Network; ROC–Receiver Operator Characteristic curve; ROCCH–ROC Convex Hull.

We constructed PLS-DA prediction models using two sample-splitting approaches commonly employed in metabolomics studies: (1) splitting the data into training (67%) and testing (33%) sets, and (2) using five-fold cross-validation (CV). First, using the 2:1 training/testing split and all 433 metabolites, we constructed several models by varying the number of PLS components included for prediction from 1 to 70 (Figure S1). This test suggested that PLS-DA analysis with a model including one component would have the best performance (as assessed by the Area under the Curve Convex Hull (AUCCH), which was 75% on the test set) on potential replication data. Because using a single training-validation partition might be sensitive to random anomalies specific to any particular split, we next implemented PLS-DA with five-fold cross-validation, and again observed model performance with varying numbers of components (see Section 4). The cross-validated AUCCHs also suggested that a model with 1 component would have the strongest performance on replication data (Figure S2). Accordingly, we proceeded by building a PLS-DA model of the full dataset with a single component. The one-component PLS-DA model achieved an AUCCH of 81% (95% confidence interval: [77.7–84.4%]) (Figure 2) on the full data, indicating high accuracy for the prediction of asthma at age three. However, a permutation test indicated a possibility of such accuracy being due to chance (*p* = 0.057: Figure S3), and that the empirical null distribution of PLS-DA accuracy had a mean 76.2% AUCCH ± 2.6%.

### 2.3. Bayesian Network Analysis

Next, we explored the predictive ability of conditional Gaussian Bayesian networks (CGBN). Typically, predictive modeling with a Bayesian network proceeds as follows. A phenotype is selected; this and the potential predictor variables are arranged in a table. These might include metabolite concentrations, demographics, clinical data, possibly or genomic variants for example. A CGBN is a type of BN that cleanly handles a mix of ordinal (typically binary) data and continuous data (typically normally distributed) within a Bayesian mathematical framework. Then a Bayesian network must be found, or discovered. Because the number of possible networks is super exponential to the number of variables, all networks cannot be considered. Heuristic algorithms are employed to identify networks likely to fit the data. Among all networks considered, the “best” network is the one that best fits the available data, as measured by the posterior probability of that data. The posterior probability is computed using Bayesian statistics and is described in more detail in Sebastiani et al. [19]. This network can then be used to identify the most likely value of a variable given the values of the other variables nearby, and in this way can be used to predict age three asthma status given metabolite information. More details are given in McGeachie et al. [20].

Following the same methodological strategy as used for PLS-DA, we used two sample-splitting approaches of (1) a 2:1 training/testing split, and (2) 5-fold cross-validation. The CGBN has one major parameter to determine: an edge-inclusion threshold for the Bayes Factor tests of statistical association. If the BF is above the threshold, an edge between two variables is added in the CGBN model. A high threshold results in a sparse model, typically with a lower risk of overfitting, but may lead to unnecessarily conservative prediction performance. As with PLS-DA, we formed a training/testing split of the dataset, and varied the BN model threshold for edge inclusion. This identified no clear edge-inclusion threshold that produced a better testing performance than the others (Figure S1), so we repeated the random process of splitting the data into training and testing portions. This ultimately produced inconclusive results. As before, we then used five-fold cross-validation to identify the best threshold for edge inclusion; with the highest cross-validated AUCCH achieved using a log BF threshold of eight (Figure S2). We then used this threshold to build a CGBN model on the full dataset. This identified a BN with 42 metabolites within the Markov Neighborhood of childhood asthma—the predictive portion of the network required to predict childhood asthma in VDAART at age three—and obtained an accuracy of 92.1% AUCCH (95% CI [90–94.1%]), indicating strong prediction (Figure 2) on the full dataset. Permutation testing indicated this level of accuracy was unlikely to be due to chance (*p* < 0.001) (Figure S4), and that the empirical null distribution of BN accuracy had mean 75.1% AUCCH ± 6.7%.

### 2.4. Comparison of Models

Both methods integrate the measurements of many metabolites into a single number that can be used to classify a subject; assigning a probability between 0.0 and 1.0 of an asthma diagnosis at age 3. When comparing the performance of the models, the classification accuracy of the Bayesian network exceeded that of the PLS-DA model (with one component). The AUCCH for the BN model was 92% on the full dataset, with a corresponding sensitivity of 82% and specificity of 84%. The one-component PLS-DA model had an AUCCH of 81% on the full dataset, with a corresponding sensitivity of 73% and specificity of 72%. The difference in AUCCHs between the PLS-DA and the BN models was statistically significant (*p* < 0.001) (Figure 2). Combined with the observations from the permutation tests, this suggests that BN model performs better at predicting age-three asthma status from the metabolites than the PLS-DA model, and this is unlikely to be due to random fluctuations in the statistical properties of the cohort or to the fitting of noise in the data.

### 2.5. Biological Interpretation of Findings

#### 2.5.1. PLS-DA 

Metabolites with influential loadings (defined as ≤−2 or ≥2) in the first component were selected for further analysis. Fifty-one metabolites had a loading in the first principal component ≤−2 and three had a loading ≥2 (Table 2).

The most strongly negative loading metabolites were glycodeoxycholate sulfate (loading: −9.1), stachydrine (−6.4), and *N*-methylproline (−6.3); the positive loading metabolites were 2-*O*-methylcytidine (2.3), and two metabolites involved in benzoate metabolism: methyl-4-hydrozybenzoate sulfate (2.4) and propyl 4-hydroxybenzoate sulfate (2.2). Half (*n* = 27) of the influential metabolites were xenobiotics; the next most represented subgroup was amino acids (*n* = 10).

#### 2.5.2. Bayesian Network Model

**Figure 3:**
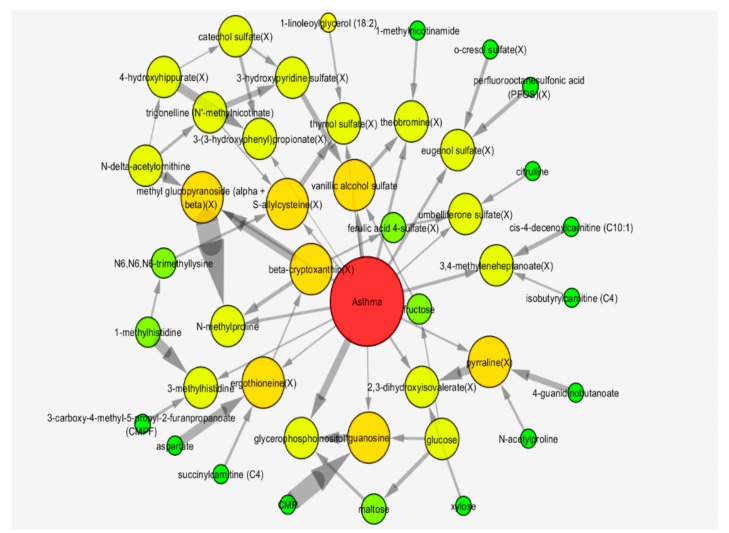
Markov neighborhood of metabolic Bayesian network for the identification of asthma at age three. Bayesian network of Year-3 asthma in VDAART. Pictured is the Markov neighborhood of the CGBN network predictive of asthma. Node size and color is proportional to degree. Directed edges represent statistical conditional dependence of the target node on the source node. Edge thickness is proportional to the statistical evidence for the edge (log Bayes Factor). Metabolite names are appended with “(X)” for those metabolites indicated as xenobiotic by Metabolon.

Forty-two metabolites were included in the Markov neighborhood of childhood asthma in the Bayesian network (Table 3, Figure 3).

Again, xenobiotics (*n* = 18) and amino acids (*n* = 11) represented the majority of the included metabolites. In particular, these were exogenous food/plant components and metabolites of the urea cycle.

Figure 3 shows edges between metabolites with thickness proportional to the statistical evidence of that edge (in log Bayes factors; a Bayesian likelihood ratio test for presence of an edge compared with its absence). We found that the strongest edge was between methyl glucopyranoside (alpha + beta) and *N*-methylproline. Since this edge is much stronger than either of the edges between these metabolites and asthma, this indicates that these two metabolites together had a much stronger effect on asthma status than either separately. Other metabolites with strong direct relationships with asthma were glycerophosphoinositol, vanillic alcohol sulfate, theobromine and eugonal sulfate. There were an additional 12 metabolites with direct edges linking them to asthma status, which were comprised mainly of xenobiotic food or plant components. (Table S2).

**Figure 4:**
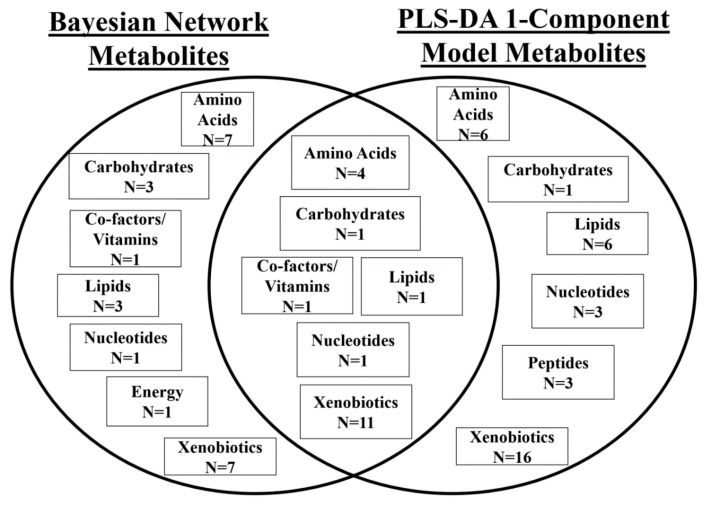
Super pathways of metabolites identified as constituent of the Bayesian network and as influential in the 1 component of the PLS-DA model, and those which are common to both methods.

Nineteen metabolites in the Bayesian network were also identified as being influential in the first PLS-DA component. These cross-over metabolites included nine xenobiotics, four amino acids, xylose, trigonelline, glycerophosphoinositol and cytidine monophosphate (CMP) (Table 4, Figure 4).

### 2.6. Sensitivity Analyses

To explore the potential impact of race, which was significantly associated with asthma status in this population, we conducted several sensitivity analyses. First, the PLS-DA and the BN models were reconstructed while including race as a potential explanatory variable together with the 433 metabolites. In this analysis, race was determined to be important in both the PLS-DA model and the CGBN model. In the PLS-DA model, race had a higher loading even than the highest-loading metabolite, and in the BN it had a direct edge with asthma. Although race was important in both models, it had little impact on the predictive performance of either; when including race, the one-component PLS-DA model had an AUCCH of 81% on the full dataset, while for the BN the AUCCH was 93% on the full dataset (Figure S5). The models also identified very similar sets of metabolites, and were consistent in terms of loadings and edge weights, with glycodeoxycholate sulfate the highest loading metabolite (PLS-DA) and the relationship between methyl glucopyranoside (alpha + beta) and *N*-methylproline demonstrating the biggest influence on asthma risk (BN).

We next investigated the power of race as an explanatory variable for asthma status by building PLS-DA and CGBN models using *only* race, with no metabolites included. The PLS-DA model using 1 PLS component and only race had 67% AUCCH (95% CI: 64.7–69.3%), and the BN model with only race had 65.5% AUCCH (95% CI: 63.2–67.8%). These results show that race is a strong indicator of age-3 asthma status in VDAART, but far from the only important indicator, and does not display as strong predictive indices as the metabolite models.

As a third investigation of the role of race, we tested the main PLS-DA and CGBN models’ ability to predict race, rather than asthma status. To accomplish this, we used a binary race attribute (White vs. non-White), and found that our CGBN (without race included) predicted the binarized race attribute with reasonable accuracy: 70.2% AUCCH (95% CI: 66.2–74.1%). Our PLS-DA model (without race) had stronger accuracy on the binarized race attribute: 76.8% AUCCH (CI: 73.2–80.3%), indicating significant performance, but statistically worse (*p* < 0.05) than predicting actual age three asthma status. These results show that both the PLS-DA and CGBN models have identified more than just the non-White VDAART participants with asthma, and that race is not driving our findings.

## 3. Discussion

To date, no definitive metabolomic profile has been identified for asthma [6]. Although many studies, particularly those utilizing PLS-DA, report impressive diagnostic and predictive indices with AUCCHs, classification rates, sensitivities, specificities and R^2^ values in excess of 90%, very few if any of these findings have been replicated in truly independent populations, and none have been translated into clinical practice [6]. One of the underlying reasons for the lack of validation of predictive models is the issue of overfitting, whereby the model fits the data so well, that in addition to identifying real disease discriminators, the model explains random variability in the dataset that has no biological meaning. This is particularly common when the number of predictors far exceeds the number of subjects, as is often the case in metabolomics. While overfitting is a problem with any machine learning methodology, in this current study, we have tried to both minimize and assess the impact of overfitting on PLS-DA and CGBN models through permutation testing. Despite these efforts, our results show that both methods are powerful enough to overfit data, since the permutation test distributions are significantly greater than 50% AUCCH. These tests showed that the PLS-DA model was on the borderline of what might be possible purely through overfitting, and this is a danger that has been noted in existing literature [8,9,10,11]. The alternative approach using Bayesian network methodology appears to be more robust, significantly outperforming the empirical null distribution. With both models, the high-powered methods and capacity for overfitting mean that the observed accuracies in the present cohort likely overestimate what would be seen on an independent replication cohort.

In this study, we compared the ability of a PLS-DA-derived plasma metabolomic signature and a novel Bayesian network derived plasma metabolomic signature to identify asthma in children aged three. Both models demonstrated extremely high accuracy, with AUCCHs in excess of 0.8 on the full dataset; however, the Bayesian network significantly out-performed the one-component PLS-DA model both in terms of accuracy, and in the likelihood of overfitting. The Bayesian network’s ability to accommodate nonlinear interactions between metabolites and relate these to the phenotype could be responsible for the increased performance, while the ability to define Bayesian priors and the Bayes Factor threshold lead to relatively parsimonious models which may explain why Bayesian networks are less susceptible to overfitting than PLS-DA.

These models reflect one of the greatest strengths of metabolomic profiling: the potential to identify biomarkers that inform on underlying etiology and pathological mechanisms. Both the PLS-DA and the BN models were enriched for metabolites and metabolic pathways with biologically interesting relationships with asthma, airway function, or other respiratory disease. Nineteen metabolites were common to the two models. This provides strong evidence for their role in asthma pathogenesis, although it must be noted, by definition, the BN identifies networks of metabolites working together which may not have a linear relationship with asthma. The fact that not all the BN metabolites are identified by the PLS-DA model should not be interpreted to mean their biological associations are not real. Common metabolites included citrulline, which has been shown to be produced at significantly higher levels from the neutrophils of asthmatics compared to non-asthmatics, with a dose-response relationship with disease severity [21]; catechol sulfate, reported to be higher in the plasma of asthmatic cases [22]; and beta-cryptoxanthin, which is inversely associated with the risk of asthma when measured in whole blood [23]. Similarly, metabolites of the gut microbiota, which have been increasingly shown to play an important role in the pathogenesis of asthma, such as 3-(3-hydroxyphenyl)propionate and 2,3-dihydroxyisovalerate ferulic acid 4-sulfate [24,25], were also identified as both influential for the PLS-DA one-component model, and as constituents of the BN. Furthermore, several of the metabolites from both the PLS-DA and BN models are involved in histidine metabolism. Histamine, a downstream product of this biological pathway, is crucial for the processes of inflammation, and has been shown to be elevated in the serum of asthmatics [26]; likely due to its role in bronchospasm and edema [27].

Both models were dominated by xenobiotics, which may not be surprising given that asthma is known to arise from complex gene-environment interactions and to be exacerbated by exogenous factors. There were also a large number of exogenous metabolites acquired through the diet. These include theobromine, a xanthine alkaloid that is known to have bronchodilator effects [28], ferulic acid 4-sulfate, a coffee metabolite that ameliorates airway inflammation in mice [29], and eugenol sulfate, which is commonly used in flavorings, but which has also been shown to have anti-asthmatic effects in mouse models [30]. 

In addition to its increased performance, the BN has the advantage of describing visually whether the constituent metabolites have direct or joint influences on asthma, and whether they may be mediated by or interacting with other metabolites within the network. This is because of BN’s ability to identify non-linear interactions; consequently, it can identify groups of metabolites working in combination to influence asthma risk that PLS-DA cannot. For example, two of the BN metabolites were aspartate and citrulline, which react to form argininosuccinate in the urea cycle. Aspartate is also involved in the nicotinate and nicotinamide metabolism pathway which is essential for the generation of coenzymes that act as precursors of redox reactions. Metabolites of this pathway, including nicotinamide are markers of inflammation and have been shown to be at higher levels in the plasma of asthmatics [22]. This is thought to be related to increased production of NAD, which would also likely lead to the altered levels of methylnicotinamide and trigonelline observed within the BN. Similarly, arginine is produced from citrulline, and it has been shown that when arginine bioavailability is low, nitric oxide (NO) production by nitric oxide synthase (for which arginine is the only substrate) is reduced. NO affects both airway tone and inflammation and has been used as a biomarker of asthma [31]. The synthesis of NO additionally produces more citrulline which can be used for recycling back to arginine. Arginine uptake can be inhibited by l-ornithine and l-lysine, and metabolites involved in the pathways that regulate levels of these metabolites, *N*-delta-acetylornithine and *N6,N6,N6*-trimethyllysine, were also identified within the BN.

In their excellent review on multivariate classification techniques in metabolomics data, Trainor et al. [11] discuss the trade-off between model interpretability versus model accuracy. However, we counter that by its nature, and as the “ome” closest to phenotype, metabolomics has the potential to do both. A growing number of classification techniques are now being applied to metabolomic data [8,11,32,33]. These include, but are not limited to, Support Vector Machine (SVM) learning models and Artificial Neural Networks (ANN), which both allow the modelling of non-linear interactions; however, these are both notoriously difficult models to interpret [33]. Similarly, Random Forests, a decision tree-based method has demonstrated high accuracy, but with somewhat limited biological interpretability [8]. Clustering approaches such as K-Nearest Neighbor, hierarchical clustering and self-organizing maps have all also been applied [32]. Furthermore, extensions to PLS-DA have been proposed, including Orthogonal PLS (OPLS), which removes the variation in the dataset unrelated to the outcome (the orthogonal variance) [34], as well as multilevel partial least squares discriminant analysis and multiblock partial least squares, which aim to improve the interpretability of the models [8,9]. On balance, Trainor et al. conclude when applied to real metabolomics data, if prediction is the goal then SVM and Random Forests perform best [11]. However, we contend that BN also represent a compelling alternative allowing for strong prediction while enabling biological interpretation and visualization of the predictors and their relationships with both each other and the outcome.

However, there were limitations to this study. In common with the majority of metabolomics studies to date, we were limited in our ability to fully explore the biology underlying these metabolomic profiles. Pathway enrichment analyses are increasing in popularity among metabolomics studies, yet metabolomics is currently less amenable to such analyses than other omic technologies such as genetics. Several excellent reviews have discussed the reasons for this in detail, and compared the currently available tools, concluding that the incompleteness of the current underlying databases and the lack of available tissue specific pathway information render their utility limited [35,36,37]. The full biological meaning of our results can only be explored with further development of metabolomics databases and enrichment tools. Given that some of the children may have been on medication for their asthma, it is possible these profiles may be driven by therapeutic regime, although we identified only one drug metabolite within our models, 4-acetylphenyl sulfate (PLS-DA model), which is not associated with asthma management. We also explored the possibility that these profiles were being driven by race. The similar results obtained when race was included as a covariate in the model suggest this is not the case, as does the greater difference in the CGBN model’s prediction of race (70.2% AUCCH) vs. the model’s prediction of asthma status (92.1% AUCCH). Finally, although we use cross-validation to identify parameters least likely to lead to over-fit models, and we show with permutation testing that the Bayesian network, and to a lesser extent the PLS-DA model, are accurate in excess of what is due to overfitting, true validation can only be achieved by replication in an independent population. In future work, it would be of interest to investigate alternative regularization methods that may provide a more complex PLS model with a smaller, but greater than one, number of components. These represent the next steps for this study.

## 4. Materials and Methods

### 4.1. The VDAART Clinical Trial: Study Participants

VDAART is a randomized double-blinded, placebo-controlled trial with centers in Boston, San Diego and St Louis, that aimed to investigate the effect of vitamin D supplementation in pregnant women on the incidence of asthma in their offspring [16]. VDAART recruited non-smoking pregnant women between 10 and 18 weeks of gestation who reported a history of asthma, eczema, or allergic rhinitis, or who had conceived the child with a man with a history of such diseases. For the remainder of their pregnancy, women were randomized 1:1 to a daily dose of 4000 IU vitamin D_3_ plus a multivitamin containing 400 IU vitamin D_3_, or a matching placebo tablet plus a multivitamin containing 400 IU vitamin D_3_. The women were followed monthly throughout their pregnancy; their offspring were then followed via telephone interviews every three months and by in-person clinic visits at age 1, 2 and 3 years. A child with a physician-diagnosis of asthma at any time during the three years of follow up was considered an asthma case. VDAART was approved by the Institutional Review Boards (IRB) of the participating Clinical Centers and the Data Coordinating Center, with pregnant women signing informed consent at the enrollment visit covering both primary and secondary analyses of data.

### 4.2. Plasma Metabolomic Profiling

Metabolomic profiling was performed on blood plasma samples extracted during the age-three visit for all children defined by a physician as asthma cases (*n* = 59), and a subset of children from the same population who did not have a physician-diagnosis of asthma during follow-up (*n* = 352). All 411 samples were stored at −80 °C until processing. Non-targeted global metabolomic profiling was conducted at Metabolon, Inc. (Durham, NC, USA), as described previously [38,39], using four ultra-performance liquid chromatography-tandem mass spectroscopy (UPLC-MS/MS) profiling platforms: (1) UPLC-MS/MS under positive ionization; (2) UPLC-MS/MS under negative ionization; (3) UPLC-MS/MS, polar platform (negative ionization); and (4) GC-MS. Metabolites were identified by their mass-to-charge ratio (*m*/*z*), retention time (rt), and through a comparison to a library of purified known standards. Data were processed in two batches sent six months apart (batch one *n* = 245; batch two *n* = 166) then scaled and merged together based on equivalence of the control groups, as follows. The second batch’s controls were scaled to the first using scaling factors making the median of the control groups in each batch equivalent. If a metabolite had a missingness of 50% or greater in either batch it was excluded from further analysis. Metabolite intensities were log transformed and those metabolites with an interquartile range of zero or with a skewness >2 or <−2 were excluded from further analyses to remove metabolites with extreme or uninformative distributions, which may bias the results.

### 4.3. Statistical Analysis

Data were bootstrap-balanced by randomly resampling the cases to obtain an equal number of cases and controls.

We used the balanced data in subsequent analyses to assure learning of an unbiased classifier. Two different methods were then utilized to build predictive models that could distinguish asthma cases from controls based on their plasma metabolomic profiles at age three: PLS-DA and Bayesian Networks. Area under the Convex Hull of the Receiver Operator Characteristic Curves (AUCCH) was used to assess predictive performance of all models, since all points on the convex hull represent realizable classification performance [40]. The difference between the AUCCHs from the PLS-DA model and the BN model were compared using the method of DeLong et al [41], following suggestions by Lasko et al. [42]. Details of each analysis are presented below.

To identify the best parameter settings for each of the classification algorithms (PLS-DA and Bayesian Networks), two strategies were employed: (1) a training (67%) and testing (33%) split of the data, and (2) five-fold cross-validation. The first was used to identify parameters of PLS-DA and Bayesian Networks that might lead to greater performance on an entirely new (replication) dataset. The second was used as a principled way to make multiple training/testing splits of the data, to limit reliance on any one particular random split of the data while still identifying parameters leading to stronger performance. Cross-validation was performed on the original 411 subjects, randomly apportioning the cases and controls separately into 5 groups, and then performing bootstrap-balancing on each fold. The parameters resulting in the best cross-validation AUCCH metrics for each strategy were then used to build PLS-DA and Bayesian network models, respectively, on the complete bootstrap-balanced dataset. The models were assessed for possible overfitting by a permutation testing, using label-shuffling with 1000 realizations. Permutation of labels was performed with the original 411 subjects, which then included new bootstrap-balancing with each permutation.

#### 4.3.1. Partial Least Squares Discriminant Analysis

PLS-DA is a supervised approach that aims to differentiate between classes (Y) in highly complex data sets, despite within-class variability in the observed variables (X). It determines the relationship between the two matrices (X and Y), by modelling their covariance structure. It finds the multidimensional direction in the X space (i.e., the metabolomic data) that explains the maximum multidimensional variance direction in the Y space (asthma status). The new subspace in X is-based upon a reduced number of factors (i.e., latent factors or components) [10,43]. The loading score provides a measure of the importance of an individual metabolite to these components. For each component of the PLS-DA models, we used a loading threshold of >2 or <−2 to identify the important contributors to that component. This is a threshold that has been shown to be a robust method of variable selection [44]. Metabolites with a loading for a component of >2 or <−2 were considered to be influential and were taken forward for further analysis. PLS-DA was performed using MATLAB version R2016a (Natick, MA, USA), with the *plsregress* function, which implements the SIMPLS algorithm [45]. Default parameter settings were used in all cases.

#### 4.3.2. Bayesian Network Analysis

To precisely model interactions between continuous-valued metabolites, a type of Bayesian network known as a conditional Gaussian Bayesian network (CGBN) (see Section 2.3) [12] was learned using the CGBayesNets [20] package in MATLAB version R2016a (MATLAB, The Mathworks Inc.; CGBayesNets, www.cgbayesnets.com). We used default parameters and algorithms to build a metabolite network predictive of asthma status at age three years in VDAART. We used the default K2 learning algorithm [46] in the CGBayesNet package throughout, which prioritizes Bayesian network (BN) edges by likelihood of association to the phenotype, and then limits potential edges to those from nodes with higher priority to those with lower priority [46]. We used the default Bayesian prior parameters (nu = 10, alpha = 10, sigma2 = 1), which control the strength of the Bayesian prior probability in each Bayesian likelihood test and default network parameter (max parents = 2), which sets a limit on the number of incoming edges to each node in the final Bayesian network Following our approach to the PLS-DA modeling, we used both (1) a hold-out set and (2) five-fold cross-validation to identify the optimal threshold for inclusion of edges in the network, measured in Bayes Factors (BF) [13]. Results from the hold-out data set were inconclusive, and therefore we focused on the cross-validation step for the Bayesian network analysis. Five-fold cross-validation was performed keeping parameter settings fixed except for varying the BF threshold from 2 to 30 by increments of 1. The cross-validation AUCCH was measured at each BF threshold. This identified the optimal BF threshold to build a CGBN on the whole dataset. The Markov Property of Bayesian networks states that only the metabolites in the Markov Neighborhood of age-three asthma are necessary to predict asthma status; these are those nodes that are parents, children, or other parents of the children of the age three asthma node. All metabolites in the Markov neighborhood, i.e., the parents, children, and other parents of those children of age-three asthma, were taken forward for further analysis.

#### 4.3.3. Permutation Tests to Assess Likelihood of Overfitting

The parameters resulting in the best cross-validation AUCCH metrics for each strategy were then used to build PLS-DA and Bayesian Network models, respectively, on the bootstrap-balanced dataset. The models were assessed for possible overfitting by permutation testing, using label-shuffling with 1000 realizations. Permutation of labels was performed with the original 411 subjects, which then included new bootstrap-balancing with each permutation.

## 5. Conclusions

In conclusion, at a time when we are still determining the optimal methodologies with which to explore metabolomic datasets, this study demonstrates the potential for Bayesian network approaches to produce robust and biologically meaningful results. This method demonstrated superior performance to PLS-DA; an approach that is commonly used in metabolomics, but has rarely identified validated profiles. This suggests BN approaches may be beneficial in the study of metabolomic datasets of other complex and chronic diseases. We present a discriminatory network for asthma in children that is characterized by several exogenous metabolites, particularly those originating from the diet, as well as dysregulation of arginine metabolism. The primacy of exogenous metabolites in our models indicates that altered metabolism of common food components is a potentially distinguishing feature of asthma.

## Figures and Tables

**Table 1:** Characteristics of the asthma cases and controls at age three.

Variable		Controls (*n* = 352)		Cases (*n* = 59)		*p*-Value
		*n*	%	*n*	%	
Gender	Male	183	52.0%	36	61.0%	0.208
	Female	169	48.0%	23	39.0%	
Race	White	119	33.8%	15	25.4%	0.003
	Black	159	45.2%	40	67.8%	
	Other	74	21.0%	4	6.8%	
Treatment Group	Placebo	182	51.7%	28	47.5%	0.576
	Intervention	170	48.3%	31	52.5%	
BMI	Mean (SD)	16.6 (2.1)		17.1 (1.9)		0.063

**Table 2:** Metabolites with an influential loading in the first PLSDA component.

Metabolite	Super Pathway	Sub Pathway	HMDB ID	Loading
glycochenodeoxycholate sulfate	Lipid	Primary Bile Acid Metabolism		−9.12
stachydrine	Xenobiotics	Food Component/Plant	HMDB04827	−6.41
*N*-methylproline	Amino Acid	Urea cycle; Arginine and Proline Metabolism		−6.31
glycolithocholate sulfate	Lipid	Secondary Bile Acid Metabolism	HMDB02639	−4.87
methyl glucopyranoside (alpha + beta)	Xenobiotics	Food Component/Plant		−4.54
theobromine	Xenobiotics	Xanthine Metabolism	HMDB02825	−4.44
cysteine s-sulfate	Amino Acid	Methionine, Cysteine, SAM and Taurine Metabolism	HMDB00731	−4.23
4-vinylguaiacol sulfate	Xenobiotics	Food Component/Plant		−4.14
taurolithocholate 3-sulfate	Lipid	Secondary Bile Acid Metabolism	HMDB02580	−4.12
3-hydroxyhippurate	Xenobiotics	Benzoate Metabolism	HMDB06116	−3.72
2,3-dihydroxyisovalerate	Xenobiotics	Food Component/Plant	HMDB12141	−3.34
4-methylcatechol sulfate	Xenobiotics	Benzoate Metabolism		−3.33
vanillic alcohol sulfate	Amino Acid	Tyrosine Metabolism		−3.25
3-(3-hydroxyphenyl)propionate	Xenobiotics	Benzoate Metabolism	HMDB00375	−3.21
p-cresol-glucuronide	Amino Acid	Tyrosine Metabolism	HMDB11686	−3.2
CMP	Nucleotide	Pyrimidine Metabolism, Cytidine containing	HMDB00095	−3.16
indolepropionate	Amino Acid	Tryptophan Metabolism	HMDB02302	−3.11
beta-cryptoxanthin	Xenobiotics	Food Component/Plant	HMDB33844	−3.08
xylose	Carbohydrate	Pentose Metabolism	HMDB00098	−3.05
tauro-beta-muricholate	Lipid	Primary Bile Acid Metabolism	HMDB00932	−3.04
5-hydroxyindoleacetate	Amino Acid	Tryptophan Metabolism	HMDB00763	−2.93
gamma-glutamylglutamate	Peptide	Gamma-glutamyl Amino Acid	HMDB11737	−2.87
ferulic acid 4-sulfate	Xenobiotics	Food Component/Plant	HMDB29200	−2.8
cinnamoylglycine	Xenobiotics	Food Component/Plant	HMDB11621	−2.71
tryptophan betaine	Amino Acid	Tryptophan Metabolism	HMDB61115	−2.7
1,2,3-benzenetriol sulfate (2)	Xenobiotics	Chemical		−2.66
catechol sulfate	Xenobiotics	Benzoate Metabolism	HMDB59724	−2.65
quinate	Xenobiotics	Food Component/Plant	HMDB03072	−2.62
inosine 5’-monophosphate (IMP)	Nucleotide	Purine Metabolism, (Hypo)Xanthine/Inosine containing	HMDB00175	−2.59
gamma-glutamylvaline	Peptide	Gamma-glutamyl Amino Acid	HMDB11172	−2.58
ergothioneine	Xenobiotics	Food Component/Plant	HMDB03045	−2.49
ribitol	Carbohydrate	Pentose Metabolism	HMDB00508	−2.49
glycerophosphoinositol	Lipid	Phospholipid Metabolism		−2.49
umbelliferone sulfate	Xenobiotics	Food Component/Plant		−2.43
pyrraline	Xenobiotics	Food Component/Plant	HMDB33143	−2.4
4-acetylphenyl sulfate	Xenobiotics	Drug		−2.37
gamma-glutamylisoleucine	Peptide	Gamma-glutamyl Amino Acid	HMDB11170	−2.36
*N*-acetylproline	Amino Acid	Urea cycle; Arginine and Proline Metabolism		−2.34
3-methoxycatechol sulfate (1)	Xenobiotics	Benzoate Metabolism		−2.32
4-vinylphenol sulfate	Xenobiotics	Benzoate Metabolism	HMDB04072	−2.29
sphinganine	Lipid	Sphingolipid Metabolism	HMDB00269	−2.29
hydantoin-5-propionic acid	Amino Acid	Histidine Metabolism	HMDB01212	−2.23
trigonelline (*N*’-methylnicotinate)	Cofactors and Vitamins	Nicotinate and Nicotinamide Metabolism	HMDB00875	−2.19
*O*-methylcatechol sulfate	Xenobiotics	Benzoate Metabolism	HMDB60013	−2.18
4-allylphenol sulfate	Xenobiotics	Food Component/Plant		−2.16
2-aminophenol sulfate	Xenobiotics	Chemical	HMDB61116	−2.14
citrulline	Amino Acid	Urea cycle; Arginine and Proline Metabolism	HMDB00904	−2.13
uridine 3’-monophosphate (3’-UMP)	Nucleotide	Pyrimidine Metabolism, Uracil containing		−2.09
3-methyl catechol sulfate (1)	Xenobiotics	Benzoate Metabolism		−2.09
3-hydroxypyridine sulfate	Xenobiotics	Chemical		−2.06
isoursodeoxycholate	Lipid	Secondary Bile Acid Metabolism	HMDB00686	−2.05
propyl 4-hydroxybenzoate sulfate	Xenobiotics	Benzoate Metabolism		2.18
2’-*O*-methylcytidine	Nucleotide	Pyrimidine Metabolism, Cytidine containing		2.33
methyl-4-hydroxybenzoate sulfate	Xenobiotics	Benzoate Metabolism		2.37

**Table 3:** Metabolites included in the Bayesian network.

Metabolite	Super Pathway	Sub Pathway	HMDB ID
1-linoleoylglycerol (18:2)	Lipid	Monoacylglycerol	
1-methylhistidine	Amino Acid	Histidine Metabolism	HMDB00001
1-methylnicotinamide	Cofactors and Vitamins	Nicotinate and Nicotinamide Metabolism	HMDB00699
2,3-dihydroxyisovalerate (X)	Xenobiotics	Food Component/Plant	HMDB12141
3-(3-hydroxyphenyl)propionate (X)	Xenobiotics	Benzoate Metabolism	HMDB00375
3-carboxy-4-methyl-5-propyl-2-furanpropanoate (CMPF)	Lipid	Fatty Acid, Dicarboxylate	HMDB61112
3-hydroxypyridine sulfate (X)	Xenobiotics	Chemical	
3-methylhistidine	Amino Acid	Histidine Metabolism	HMDB00479
3,4-methyleneheptanoate (X)	Xenobiotics	Food Component/Plant	
4-guanidinobutanoate	Amino Acid	Guanidino and Acetamido Metabolism	HMDB03464
4-hydroxyhippurate (X)	Xenobiotics	Benzoate Metabolism	HMDB13678
aspartate	Amino Acid	Alanine and Aspartate Metabolism	HMDB00191
beta-cryptoxanthin (X)	Xenobiotics	Food Component/Plant	HMDB33844
catechol sulfate (X)	Xenobiotics	Benzoate Metabolism	HMDB59724
cis-4-decenoylcarnitine (C10:1)	Lipid	Fatty Acid Metabolism (Acyl Carnitine)	
citrulline	Amino Acid	Urea cycle; Arginine and Proline Metabolism	HMDB00904
CMP	Nucleotide	Pyrimidine Metabolism, Cytidine containing	HMDB00095
Ergothioneine (X)	Xenobiotics	Food Component/Plant	HMDB03045
eugenol sulfate (X)	Xenobiotics	Food Component/Plant	
ferulic acid 4-sulfate (X)	Xenobiotics	Food Component/Plant	HMDB29200
fructose	Carbohydrate	Fructose, Mannose and Galactose Metabolism	HMDB00660
glucose	Carbohydrate	Glycolysis, Gluconeogenesis, and Pyruvate Metabolism	HMDB00122
glycerophosphoinositol	Lipid	Phospholipid Metabolism	
guanosine	Nucleotide	Purine Metabolism, Guanine containing	HMDB00133
isobutyrylcarnitine (C4)	Amino Acid	Leucine, Isoleucine and Valine Metabolism	HMDB00736
maltose	Carbohydrate	Glycogen Metabolism	HMDB00163
methyl glucopyranoside (alpha + beta) (X)	Xenobiotics	Food Component/Plant	
*N*-acetylproline	Amino Acid	Urea cycle; Arginine and Proline Metabolism	
*N*-delta-acetylornithine	Amino Acid	Urea cycle; Arginine and Proline Metabolism	
*N*-methylproline	Amino Acid	Urea cycle; Arginine and Proline Metabolism	
*N*6,*N*6,*N*6-trimethyllysine	Amino Acid	Lysine Metabolism	HMDB01325
o-cresol sulfate (X)	Xenobiotics	Benzoate Metabolism	
perfluorooctanesulfonic acid (PFOS) (X)	Xenobiotics	Chemical	HMDB59586
Pyrraline (X)	Xenobiotics	Food Component/Plant	HMDB33143
S-allylcysteine (X)	Xenobiotics	Food Component/Plant	HMDB34323
succinylcarnitine (C4)	Energy	TCA Cycle	HMDB61717
Theobromine (X)	Xenobiotics	Xanthine Metabolism	HMDB02825
thymol sulfate (X)	Xenobiotics	Food Component/Plant	HMDB01878
trigonelline (*N*’-methylnicotinate)	Cofactors and Vitamins	Nicotinate and Nicotinamide Metabolism	HMDB00875
umbelliferone sulfate (X)	Xenobiotics	Food Component/Plant	
vanillic alcohol sulfate	Amino Acid	Tyrosine Metabolism	
xylose	Carbohydrate	Pentose Metabolism	HMDB00098

**Table 4:** Metabolites that were identified as influential in the PLS-DA first component and were included in the Bayesian Network.

Metabolite	Super Pathway	Sub Pathway	HMDB ID
vanillic alcohol sulfate	Amino Acid	Tyrosine Metabolism	
citrulline	Amino Acid	Urea cycle; Arginine and Proline Metabolism	HMDB00904
*N*-acetylproline	Amino Acid	Urea cycle; Arginine and Proline Metabolism	
*N*-methylproline	Amino Acid	Urea cycle; Arginine and Proline Metabolism	
xylose	Carbohydrate	Pentose Metabolism	HMDB00098
trigonelline (*N’*-methylnicotinate)	Cofactors and Vitamins	Nicotinate and Nicotinamide Metabolism	HMDB00875
Glycerophosphoinositol	Lipid	Phospholipid Metabolism	
CMP	Nucleotide	Pyrimidine Metabolism, Cytidine containing	HMDB00095
3-(3-hydroxyphenyl)propionate	Xenobiotics	Benzoate Metabolism	HMDB00375
catechol sulfate	Xenobiotics	Benzoate Metabolism	HMDB59724
2,3-dihydroxyisovalerate	Xenobiotics	Food Component/Plant	HMDB12141
beta-cryptoxanthin	Xenobiotics	Food Component/Plant	HMDB33844
ergothioneine	Xenobiotics	Food Component/Plant	HMDB03045
ferulic acid 4-sulfate	Xenobiotics	Food Component/Plant	HMDB29200
methyl glucopyranoside (alpha + beta)	Xenobiotics	Food Component/Plant	
pyrraline	Xenobiotics	Food Component/Plant	HMDB33143
umbelliferone sulfate	Xenobiotics	Food Component/Plant	
theobromine	Xenobiotics	Xanthine Metabolism	HMDB02825
3-hydroxypyridine sulfate	Xenobiotics	Chemical	

## Supplementary Materials

The following are available online at http://www.mdpi.com/2218-1989/8/4/68/s1, Table S1: Super Pathway and Sub Pathway of 433 Metabolites included in the analyses, Table S2: Edge Weights between a pair of nodes in the BN, Table S3: Metabolites that were identified as influential in the PLS-DA first component and were included in the Bayesian Network, Figure S1: Hold-out testing of PLS-DA and BN models, Figure S2: Permutation test of 1 PLS-DA Component, Figure S3: Cross-validation of Bayesian Network and PLS-DA for Asthma in VDAART, Figure S4: Permutation test of Bayesian Network, Figure S5 VDAART Metabolite Asthma Prediction by BN (AUCCH = 0.926) and PLS-DA (AUCCH = 0.803) including race on the full dataset.
