# Supplementary material for: Partial Least Squares Discriminant Analysis and Bayesian Networks for Metabolomic Prediction of Childhood Asthma
Source: Metabolites. 2018 Oct 23;8(4):68. doi: 10.3390/metabo8040068 (PMC6316795; doi:10.3390/metabo8040068)
Supplement: Supplementary file 1 [file metabolites-08-00068-s001.pdf]

Supplementary Material

Table S1: Super Pathway and Sub Pathway of 433 Metabolites included in the analyses

Super Pathway	Sub Pathway	n	%
Lipids	Fatty Acid Metabolism	39	9.00%
Lipids	Sphingolipid Metabolism	25	5.80%
Lipids	Fatty Acid	22	5.10%
Lipids	Secondary Bile Acid Metabolism	12	2.80%
Lipids	Primary Bile Acid Metabolism	8	1.80%
Lipids	Phospholipid Metabolism	7	1.60%
Lipids	Endocannabinoid	5	1.20%
Lipids	Sterol	4	0.90%
Lipids	Androgenic Steroids	3	0.70%
Lipids	Medium Chain Fatty Acid	3	0.70%
Lipids	Carnitine Metabolism	2	0.50%
Lipids	Corticosteroids	2	0.50%
Lipids	Glycerolipid Metabolism	2	0.50%
Lipids	Monoacylglycerol	2	0.50%
Lipids	Progestin Steroids	2	0.50%
Lipids	Fatty Acid Synthesis	1	0.20%
Lipids	Inositol Metabolism	1	0.20%
Lipids	Ketone Bodies	1	0.20%
Lipids	Mevalonate Metabolism	1	0.20%
Amino Acid	Leucine, Isoleucine and Valine Metabolism	26	6.00%
Amino Acid	Tyrosine Metabolism	16	3.70%
Amino Acid	Urea cycle; Arginine and Proline Metabolism	16	3.70%
Amino Acid	Tryptophan Metabolism	14	3.20%
Amino Acid	Methionine, Cysteine, SAM and Taurine Metabolism	12	2.80%
Amino Acid	Glycine, Serine and Threonine Metabolism	9	2.10%
Amino Acid	Histidine Metabolism	9	2.10%
Amino Acid	Glutamate Metabolism	8	1.80%
Amino Acid	Lysine Metabolism	8	1.80%
Amino Acid	Alanine and Aspartate Metabolism	5	1.20%
Amino Acid	Glutathione Metabolism	5	1.20%
Amino Acid	Polyamine Metabolism	5	1.20%
Amino Acid	Phenylalanine Metabolism	4	0.90%
Amino Acid	Creatine Metabolism	3	0.70%
Amino Acid	Guanidino and Acetamido Metabolism	1	0.20%

Supplementary Table 1: *cont*

Super Pathway	Sub Pathway	n	%
Xenobiotics	Food Component/Plant	23	5.30%
Xenobiotics	Benzoate Metabolism	16	3.70%
Xenobiotics	Chemical	13	3.00%
Xenobiotics	Bacterial/Fungal	2	0.50%
Xenobiotics	Xanthine Metabolism	2	0.50%
Xenobiotics	Drug	1	0.20%
Nucleotide	Pyrimidine Metabolism, Cytidine containing	14	3.20%
Nucleotide	Purine Metabolism	13	3.00%
Peptide	Gamma-glutamyl Amino Acid	15	3.50%
Peptide	Dipeptide	4	0.90%
Peptide	Acetylated Peptides	3	0.70%
Cofactors and Vitamins	Hemoglobin and Porphyrin Metabolism	5	1.20%
Cofactors and Vitamins	Nicotinate and Nicotinamide Metabolism	5	1.20%
Cofactors and Vitamins	Tocopherol Metabolism	4	0.90%
Cofactors and Vitamins	Ascorbate and Aldarate Metabolism	3	0.70%
Cofactors and Vitamins	Pantothenate and CoA Metabolism	1	0.20%
Cofactors and Vitamins	Vitamin A Metabolism	1	0.20%
Cofactors and Vitamins	Vitamin B6 Metabolism	1	0.20%
Carbohydrate	Glycolysis, Gluconeogenesis, and Pyruvate Metabolism	6	1.40%
Carbohydrate	Pentose Metabolism	5	1.20%
Carbohydrate	Fructose, Mannose and Galactose Metabolism	3	0.70%
Carbohydrate	Aminosugar Metabolism	2	0.50%
Carbohydrate	Glycogen Metabolism	2	0.50%
Energy	TCA Cycle	5	1.20%
Energy	Oxidative Phosphorylation	1	0.20%

Table S2: Edge Weights between a pair of nodes in the BayesNet

**Source Node**	**Sink Node**	**Edge Weight**
methyl glucopyranoside (alpha + beta)	N-methylproline	228.9
CMP	guanosine	168.14
1-methylhistidine	3-methylhistidine	95.72
beta-cryptoxanthin	methyl glucopyranoside (alpha + beta)	88.63
aspartate	ergothioneine	81.13
4-hydroxyhippurate	3-(3-hydroxyphenyl)propionate	80.26
pyrraline	2,3-dihydroxyisovalerate	72.58
**Asthma**	glycerophosphoinositol	70.41
guanosine	glycerophosphoinositol	59.26
N-delta-acetylornithine	methyl glucopyranoside (alpha + beta)	57.85
4-guanidinobutanoate	pyrraline	53.39
S-allylcysteine	thymol sulfate	50.99
trigonelline (N'-methylnicotinate)	3-hydroxypyridine sulfate	50.85
3-hydroxypyridine sulfate	vanillic alcohol sulfate	48.14
cis-4-decenoylcarnitine (C10:1)	3,4-methyleneheptanoate	38.64
perfluorooctanesulfonic acid (PFOS)	eugenol sulfate	38.57
**Asthma**	vanillic alcohol sulfate	38.49
glucose	maltose	37.16
beta-cryptoxanthin	N-methylproline	35.85
vanillic alcohol sulfate	theobromine	35.5
o-cresol sulfate	eugenol sulfate	34.86
catechol sulfate	3-(3-hydroxyphenyl)propionate	31.11
**Asthma**	theobromine	29.77
**Asthma**	eugenol sulfate	28.86
maltose	glycerophosphoinositol	28.26
**Asthma**	3,4-methyleneheptanoate	28.22
N-delta-acetylornithine	trigonelline (N'-methylnicotinate)	26.9

Table S2: *cont*

Source Node	Sink Node	Edge Weight
**Asthma**	N-methylproline	25.62
1-methylnicotinamide	theobromine	25.61
succinylcarnitine (C4)	ergothioneine	25.54
N-acetylproline	pyrraline	24.37
glucose	guanosine	24.08
3-carboxy-4-methyl-5-propyl-2-furanpropanoate (CMPF)	3-methylhistidine	23.95
**Asthma**	2,3-dihydroxyisovalerate	22.52
catechol sulfate	3-hydroxypyridine sulfate	21.52
N6,N6,N6-trimethyllysine	S-allylcysteine	21.33
**Asthma**	methyl glucopyranoside (alpha + beta)	21.25
ferulic acid 4-sulfate	umbelliferone sulfate	20.72
citrulline	umbelliferone sulfate	20.36
1-linoleoylglycerol (18:2)	thymol sulfate	20.06
1-methylhistidine	N6,N6,N6-trimethyllysine	19.53
beta-cryptoxanthin	ferulic acid 4-sulfate	18.96
isobutyrylcarnitine (C4)	3,4-methyleneheptanoate	18.85
**Asthma**	pyrraline	18.35
fructose	vanillic alcohol sulfate	16.48
**Asthma**	S-allylcysteine	16.35
**Asthma**	umbelliferone sulfate	15.94
**Asthma**	3-methylhistidine	15.66
**Asthma**	thymol sulfate	15.5
ergothioneine	beta-cryptoxanthin	15.08
**Asthma**	ergothioneine	14.79
trigonelline (N'-methylnicotinate)	S-allylcysteine	14.71
glucose	fructose	13.56
**Asthma**	guanosine	13.56
4-hydroxyhippurate	catechol sulfate	12.22
N-delta-acetylornithine	4-hydroxyhippurate	11.31
**Asthma**	3-(3-hydroxyphenyl)propionate	8.15

The starting node is known as the "Source" and the node the edge points to is known as the "sink"

Figure S1: Hold-out testing of PLS-DA and BN models

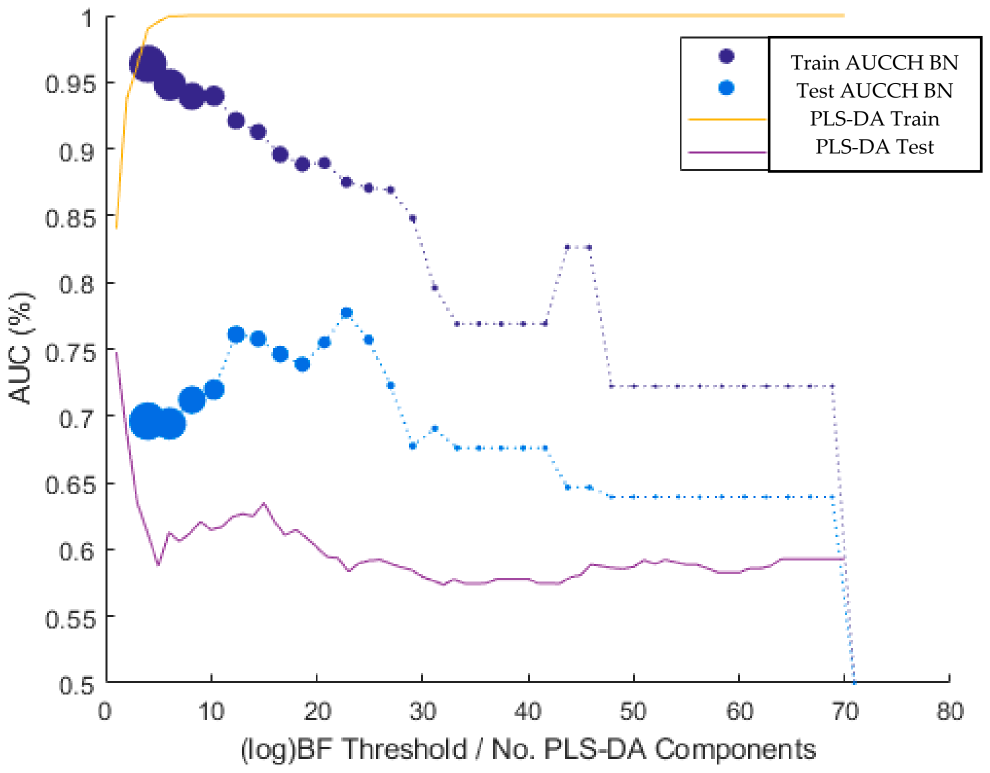

Data were split into a hold-out testing set (33%) and a training set (67%). PLS-DA was trained on the training set for each possible number of components, 1 to 70. Components are plotted along the x-axis. PLS-DA prediction on the testing set is measured in AUCCH and shown in purple. CGBNs were learned on the training set using different edge thresholds (log Bayes Factors, BF) for including edges in the network. Training performance and testing performance are shown for each BN, with total network size proportional to the size of the dot. BFs and Number of PLS-DA components are shown on the same axis, in the same direction.

Figure S2: Cross Validation of accuracy of PLS-DA and Bayesian Network for Asthma in VDAART

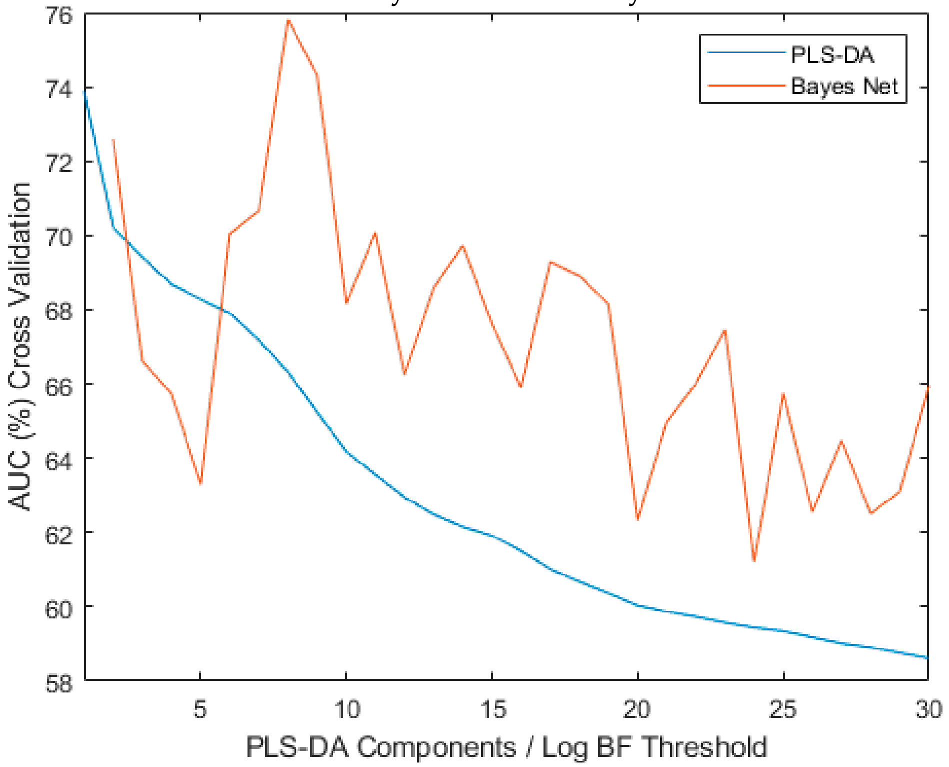

Using five-fold cross validation, Bayesian Networks and PLS-DA were assessed using different edge-threshold inclusion parameters (in log Bayes Factor, BF), and PLS-DA components, shown on the x-axis.

Figure S3: Permutation test of 1 PLS-DA Component. AUCCH=0.810, p=0.057

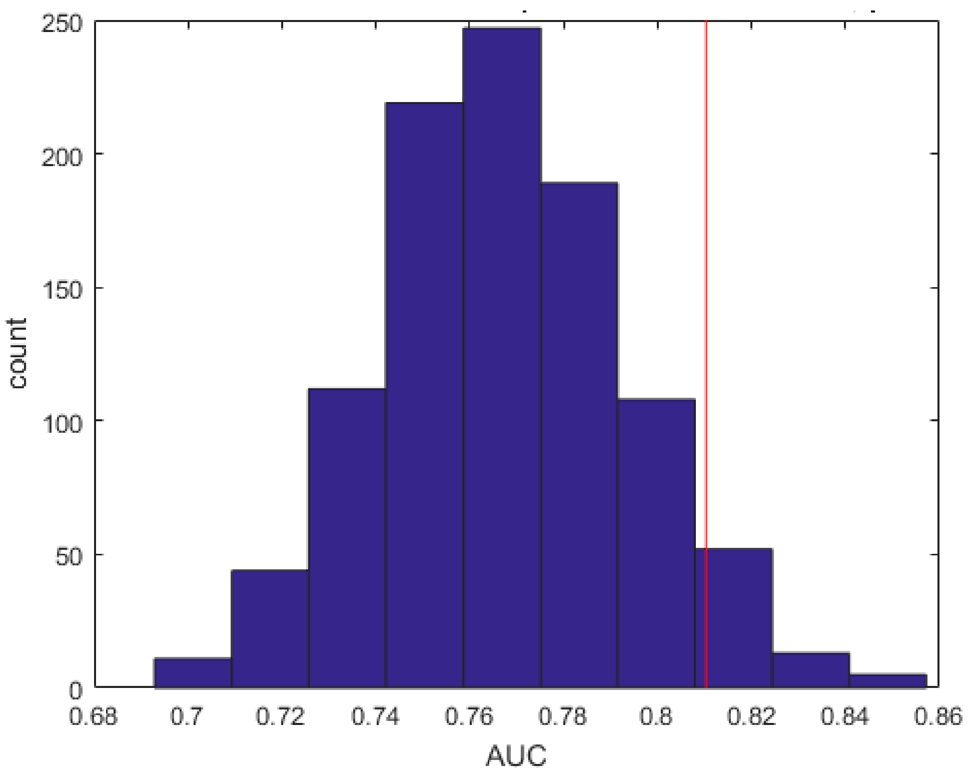

The empirical distribution of possible AUCCHs for PLS-DA models with 1 component from 1000 random realizations of the VDAART data, with the asthma status (case / control) randomly permuted in each. The AUCCH of the 1-component PLS-DA model on the true data is shown as a red line. This test indicates a small likelihood of obtaining similar accuracy by chance (p = 0.057).

Figure S4: Permutation test of Bayesian Network; AUCCH=0.92, p<0.001

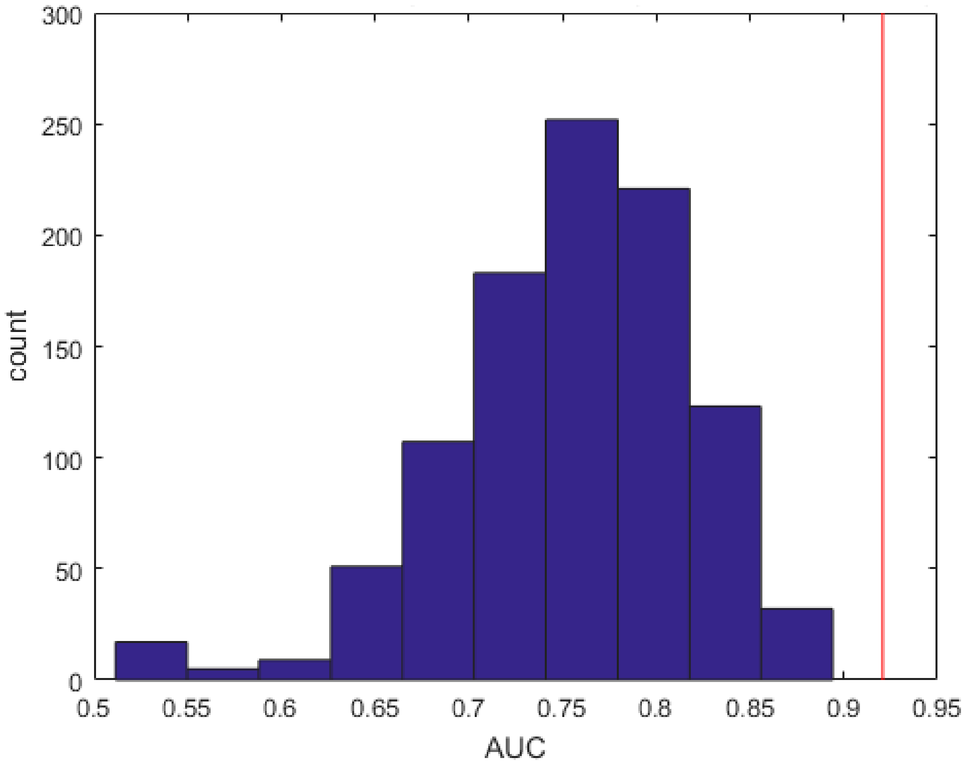

The empirical distribution of possible AUCCHs for Bayesian Network models from 1000 random realizations of the VDAART data, with the asthma status (case / control) randomly permuted in each. The AUCCH of the BN model on the true data is shown as a red line (AUCCH = 0.92). This test indicates a very small likelihood of obtaining similar accuracy by chance ($p < 0.001$).

Figure S5 VDAART Metabolite Asthma Prediction by BN (AUCCH=0.926) and PLS-DA (AUCCH=0.803) including Race

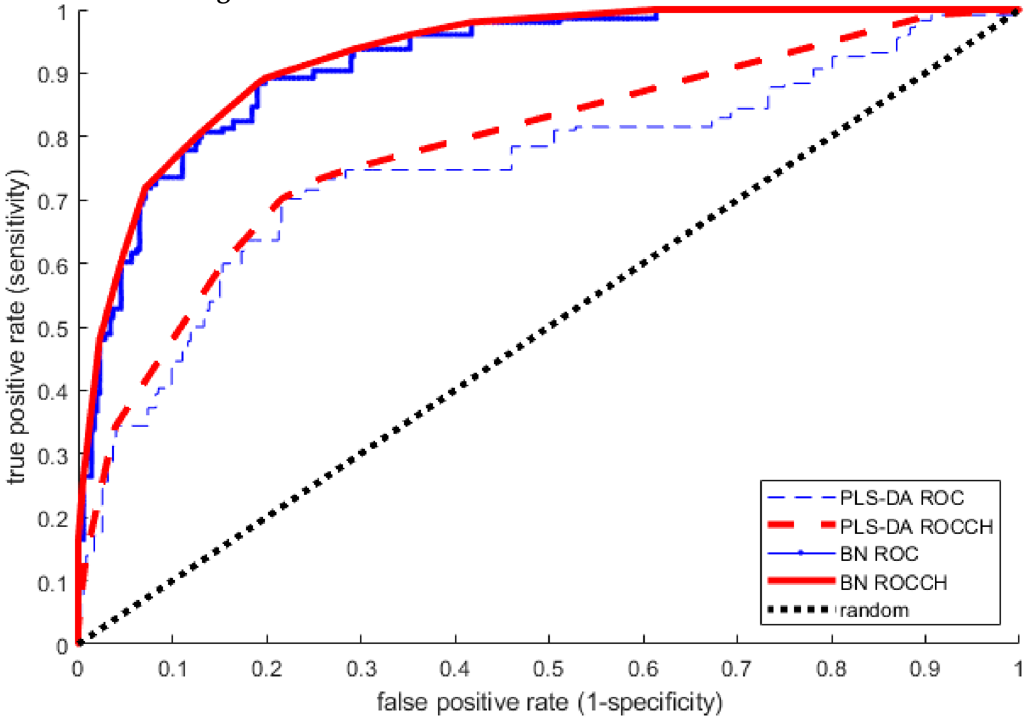

The Discrimination of asthma at age three based on plasma metabolomic profiles by PLS-DA (AUCCH=0.803) and by Bayesian network (AUCCH=0.926) including Race on the full dataset. AUCCH- Area under the curve convex hull. BN – Bayesian Network. ROC – Receiver Operator Characteristic curve

ROCCH – ROC Convex Hull.
